# Hedgehog signaling is controlled by Rac1 activity

**DOI:** 10.7150/thno.67702

**Published:** 2022-01-01

**Authors:** Chao Tang, Ximei Wu, Qianlei Ren, Minli Yao, Shouying Xu, Ziyi Yan

**Affiliations:** 1 National Clinical Research Center for Child Health of the Children's Hospital, Zhejiang University School of Medicine, Hangzhou, 310052, China; 2 Department of Pharmacology, School of Medicine, Zhejiang University, Hangzhou, 310058, China

**Keywords:** Rac1, Vav2, PAK1, IFT, KIF, Hedgehog, Gli

## Abstract

**Rationale:** The nuclear translocation of transcriptional factor Gli is indispensable for Hedgehog (Hh) signaling activation, whose deregulation causes cancer progressions; however, the mechanisms governing Gli nuclear translocation are poorly understood. Here, we report that the Gli translocation in response to Hh requires Rac1 activation.

**Methods:** C3H10T1/2 cell line and mouse embryonic fibroblasts were used to explore the molecular mechanisms underlying Rac1 activity in regulation of Hh signaling transduction. Transgenic mouse strains and human medulloblastoma (MB) tissue samples were utilized to examine the role of Rac1 in Hh-directed limb bud development and MB progression.

**Results:** We show that upon the binding of Hh to receptor Patched1 (Ptch1), receptor Smoothened (Smo) dissociates from Ptch1 and binds to Vav2, resulting in the increased phosphorylation levels of Vav2 at Y172, which further activates Rac1. The role of Rac1 is dependent on the regulation of phosphorylation levels of KIF3A at S689 and T694, which in turn affects IFT88 stability and subsequently dampens SuFu-Gli complex formation, leading to the release of Gli from the complex and the consequent translocation of Gli into the nucleus. Moreover, Vav2 phospho-Y172 levels are up-regulated in *GFAP-Cre;SmoM2^+/-^* mouse cerebellum and human Shh type MB tissues, whereas deficiency of *Rac1* in mouse embryonic limb bud ectoderm (*Prx1-Cre;Rac1^f/f^*) impedes Hh activation by disruption of Gli nuclear translocation.

**Conclusion:** Together, our results uncover the Rac1 activation and the subsequent Gli translocation as a hitherto uncharacterized mechanism controlling Hh signaling and may provide targets for therapeutic intervention of this signaling pathway.

## Introduction

The Hedgehog (Hh) signaling pathway is one of the key regulators of intercellular communication in development within metazoans, and is critical in homeostasis and regeneration as well as in tumoregenesis, such as medulloblastoma (MB) and basal cell carcinoma (BCC) 1-5. The *Hh* gene was initially discovered in *Drosophila* based on the phenotype of fly larvae that lack *Hh*, with ectopic denticles resembling a hedgehog 6. There are three mammalian Hh proteins, including Sonic Hedgehog (Shh), Indian Hedgehog (Ihh) and Desert Hedgehog (Dhh), among which Shh is the most broadly expressed and is thought to be responsible for the major effects on development. Unlike other core developmental signaling pathways, vertebrate Hh signaling is completely dependent on a highly specialized organelle, the primary cilium 7. In the absence of Hh ligand, receptor patched-1 (Ptch1) represses receptor smoothened (Smo) activity and thereby the transcriptional factor of Gli family is associated with Suppressor of Fused (SuFu), and undergoes phosphorylation by protein kinases such as protein kinase A (PKA), glycogen synthase kinase 3β (GSK3β) and casein kinase 1(CK1), and subsequently proteolytical cleavage into its repressor within the primary cilium, resulting in inactivation of Hh signaling 8,9. However, binding of Hh to Ptch1 leads to relieving the inhibition of Smo, resulting in the rapid dissociation of the SuFu-Gli complex, thus allowing Gli to enter the nucleus and transactivate the target genes including *Cyclin D*, *Cyclin E*, *c-Myc* as well as *Ptch1* and *Gli1* 9.

Although nuclear localization of Gli in response to Hh is essential for Hh signaling transduction, mechanisms controlling this process are not well understood. Previous reports suggested that activation of Hh causes separation of Gli from Sufu-Gli complex at primary cilium basal body, and the dissociated Gli is thereby carried by intraflagellar transport complexes B (IFT-B) that is powered by heterotrimeric kinesin-2 complex, consisting of KIF3A, KIF3B, and KIF associated protein 3 (KAP3), to the cilium tip, and is subsequently carried back by intraflagellar transport complexes A (IFT-A) to the basal body powered by cytoplasmic dynein 2 motor made up of a heavy chain, an intermediate chain, a light intermediate chain and several light chains, thus resulting in the translocation of Gli from primary cilium basal body to cytoplasm and further into nucleus 10-12.

The Rho family of small GTPases regulates cytoskeleton and transcription by virtue of cycling between inactive GDP-bound and active GTP-bound forms 13. Members of the family, including RhoA, Rac1 and Cdc42, have been shown to participate in the key developmental signaling pathways, such as the canonical Wnt signaling, the noncanonical Wnt signaling, the Hippo signaling as well as the Notch signaling 14-17, indicating the essential role of the Rho family of small GTPases in development. Moreover, the activitiy of RhoA and Cdc42 has been found to be altered by Hh activation 18,19, suggesting the role of Rho family of small GTPases in Hh signaling transduction.

Here, we report that the Rac1 activation is a critical component of Hh signaling. Specifically, we show that upon Rac1 activaton, Y172-phospho-Vav2 through PAK1 phosphorylates KIF3A on critical residues, which in turn binds to and inhibits IFT88 degradation and subsequently dampens SuFu-Gli complex formation, leading to the release of Gli from the SuFu-Gli complex and translocation into the nucleus. In addition, we present the evidence that hyper-activation of Smo in mouse cerebella (*GFAP-Cre;SmoM2^+/-^*) results in the increased phosphorylation levels of Vav2 and PAK1, and similarly, the phosphorylation levels of Vav2 are up-regulated in human Shh type MB tissues, whereas* Rac1* loss in mouse embryonic limb bud ectoderm (*Prx1-Cre;Rac1^f/f^*) suppresses Hh activation with the failure in Gli nuclear translocation.

## Results

### Rac1 activation promotes Hh signaling

**Figure 1:**
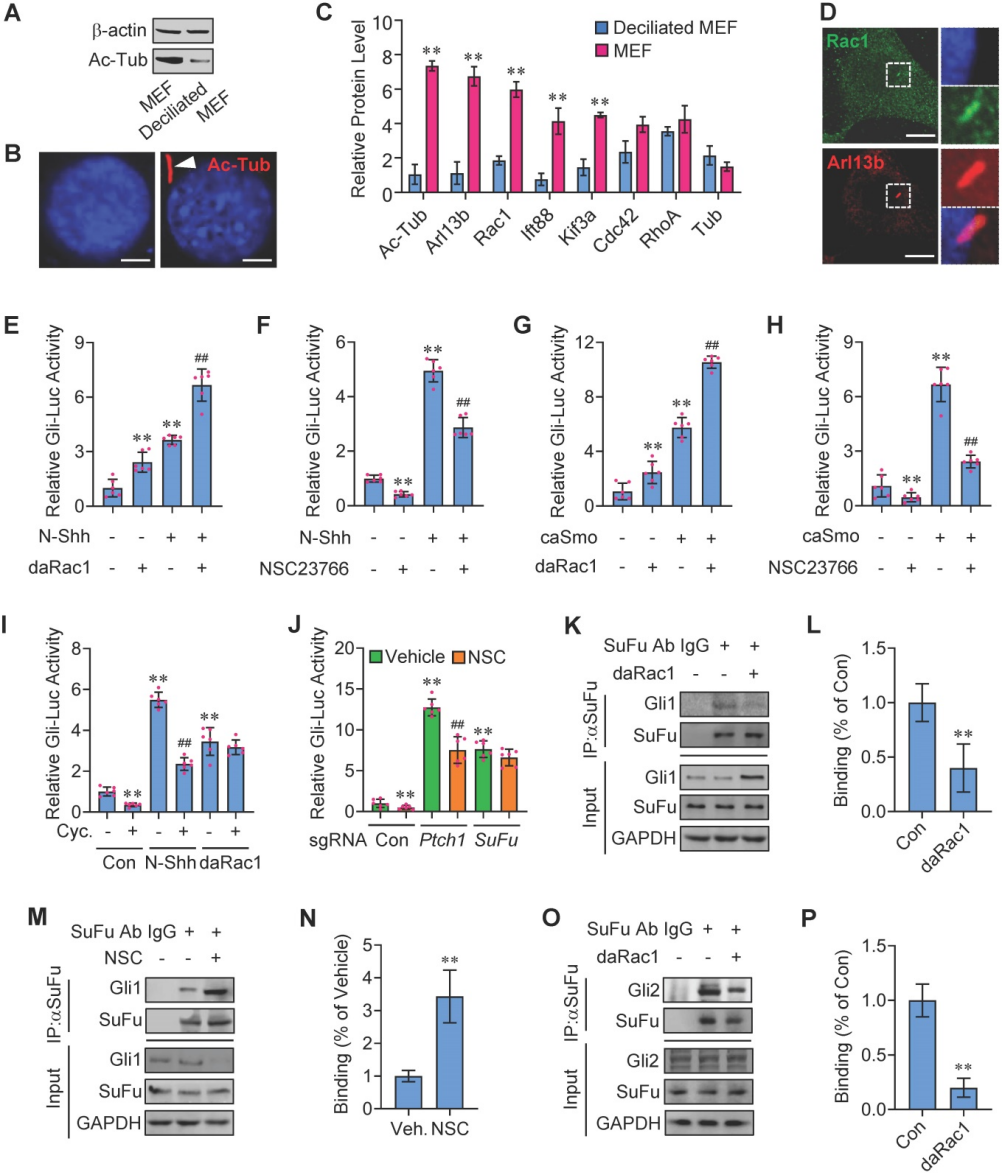
** Potentiation of Hh signaling by Rac1 activation. (A)** Total cellular membrane lysates from MEFs in high densities and deciliated MEFs in low densities were subjected to immunoblotting. The membrane lysates of MEFs from high densities are enriched for the ciliary component acetylated tubulin (Ac-Tub). **(B)** Immunofluorescence staining (red) for Ac-Tub in MEFs and deciliated MEFs. Nuclei were counterstained by DAPI. Bar, 20 μm. **(C)** LC-MS/MS quantitation of cilia markers and Rho family of small GTPases extracted from deciliated MEFs and MEFs. N=3. **(D)** Immunofluorescence staining for Rac1 in MEF. Primary cilia were indicated by Arl13b staining. Nuclei were counterstained by DAPI. Bar, 15 μm. **(E)** C3H10T1/2 cells were transiently transfected with a Gli luciferase reporter together with daRac1 and cultured with or without N-Shh at 100 ng/ml for 24 h. Total cell lysates were subjected to luciferase assay. **(F)** C3H10T1/2 cells were transiently transfected with a Gli luciferase reporter and cultured with or without N-Shh at 100 ng/ml or NSC23766 at 10 μg/ml for 24 h. Total cell lysates were subjected to luciferase assay. **(G)** C3H10T1/2 cells were transiently transfected with a Gli luciferase reporter together with daRac1 and caSmo and cultured for 24 h. Total cell lysates were subjected to luciferase assay. **(H)** C3H10T1/2 cells were transiently transfected with a Gli luciferase reporter together with caSmo and cultured with or without NSC23766 at 10 μg/ml for 24 h. Total cell lysates were subjected to luciferase assay. **(I)** C3H10T1/2 cells were transiently transfected with a Gli luciferase reporter together with daRac1 and cultured with or without Cyclopamine (Cyc.) at 5 μM for 24 h. Total cell lysates were subjected to luciferase assay. **(J)** *Ptch1*-knockout (sgRNA-*Ptch1*), *SuFu*-knockout (sgRNA-*SuFu*) or control (sgRNA-Con) C3H10T1/2 cells were transiently transfected with a Gli luciferase reporter for 48 h and cultured with or without NSC23766 (NSC) at 10 μg/ml for 24 h. Total cell lysates were subjected to luciferase assay. **(K)** C3H10T1/2 cells were transfected with daRac1. Total cell lysates (Input) and anti-SuFu immunoprecipitates (IP, SuFu Ab, +) from total cell lysates were analyzed by immunoblotting with anti-SuFu and anti-Gli1 antibodies. IgG was used as a negative control for IP. **(L)** Quantification via densitometry (n=3) and statistical analysis of Gli1 bands of (K). **(M)** C3H10T1/2 cells were cultured with NSC23766 (NSC) at 10 μg/ml for 24 h. Total cell lysates (Input) and anti-SuFu immunoprecipitates (IP, SuFu Ab, +) from total cell lysates were analyzed by immunoblotting with anti-SuFu and anti-Gli1 antibodies. IgG was used as a negative control for IP. **(N)** Quantification via densitometry (n=3) and statistical analysis of Gli1 bands of (M). **(O)** C3H10T1/2 cells were transfected with or without daRac1. Total cell lysates (Input) and anti-SuFu immunoprecipitates (IP, SuFu Ab, +) from total cell lysates were analyzed by immunoblotting with anti-SuFu and anti-Gli2 antibodies. IgG was used as a negative control for IP. **(P)** Quantification via densitometry (n=3) and statistical analysis of Gli2 bands of (O). Protein abundance normalized to GAPDH, respectively. *p < 0.05; **, ^##^*p* < 0.01; n=6 in (E-J), error bar, SD.

The primary cilia are essential for Hh signaling transduction and the growth of primary cilia is regulated by cellular confluency 1,20-22. To identify new components involved in Hh signaling transduction, we isolated the mouse embryonic fibroblasts (MEFs) and seeded them in different densities to make MEFs deciliated or ciliated and biochemically extracted total membrane proteins from the deciliated MEFs in low densities as well as the ciliated MEFs in high densities (Figure 1A-B). Data of liquid chromatography-tandem mass spectrometry (LC-MS/MS) with normalization to protein content revealed that, compared with the deciliated MEFs, the ciliated MEFs are enriched in acetylated tubulin (Ac-Tub) and Arl13b, two markers for primary cilia 23,24, and IFT88 and KIF3A as well, two molecules pivotal to the maintenance and function of primary cilia 25,26 (Figure 1C). Intriguingly, among the three members in Rho family of small GTPases 27, only enrichment of Rac1 but not Cdc42 or RhoA was observed in the ciliated MEFs, compared with that in deciliated MEFs (Figure 1C), and the enrichment of Rac1 in MEF primary cilia was additionally verified by immunnofluorescent staining (Figure 1D), indicating that Rac1 may affect Hh signaling.

To investigate the potential roles of Rac1 in Hh signaling regulation, we transfected an established 8 × Gli-binding site-luciferase (Gli-Luc) reporter construct into C3H10T1/2 cells, a cell line of mouse embryonic fibroblasts that is widely used for Hh signaling investigation 28,29, and performed the Gli-Luc reporter assays. Recombinant mouse Shh N-terminus protein (N-Shh) robustly induced the Gli-Luc activities, which were further potentiated by the overexpression of a constitutively active form of Rac1 (V12-Rac1, daRac1) (Figure 1E). Conversely, either knockdown of Rac1 by Rac1-siRNA or inactivation of Rac1 by a selective chemical inhibitor NSC23766 30 significantly reduced the Gli-Luc activities in both the presence and absence of N-Shh (Figure 1F, Figure S1A). In addition, the effect of Rac1 activity on Hh signaling was tested by overexpression of a wild-type Rac1 (Rac1-WT), a daRac1 (Rac1-DA) or a dominant negative form of Rac1 (dnRac1, Rac1-DN, N17-Rac1) in the *Rac1*-knockout (*Rac1*^KO^) C3H10T1/2 cells, and the data showed that, overexpression of daRac1 markedly induced the Gli-Luc activities, whereas overexpression of dnRac1 significantly suppressed the Gli-Luc reporter activities, compared with the WT group, respectively (Figure S1B).

To further confirm the positive regulation of Rac1 activation in Hh signaling, we transfected C3H10T1/2 cells with a constitutively active form of Smoothened (caSmo) to activate Hh-Smo downstream signaling. Similarly, caSmo significantly upregulated the Gli-Luc activities that were amplified by the existence of daRac1, whereas either Rac1 siRNA or NSC23766 not only down-regulated Gli-Luc activities but also restored the caSmo-enhanced Gli-Luc activities (Figure 1G-H, Figure S1C). Thus, these data demonstrated that Rac1 activation promotes Hh signaling. However, unlike Rac1, neither overexpression nor knockdown of its closely related isoforms, Rac2 and Rac3 31,32, whose knockdown efficiency was comfirmed by western blot analysis, affected the Gli-Luc activities in C3H10T1/2 cells (Figure S1D-H), and we speculated that it might be due to the variations in the carboxy-terminal (Ct) sequence among the three Rac isoforms (Figure S1I). Unexpectedly but intriguingly, though cyclopamine (Cyc.), a Smo antagonist 33, obviously inhibited Gli-Luc activities in the presence or absence of N-Shh (Figure 1I), it had no effect on the daRac1-induced Gli-Luc activities (Figure 1I), indicating the activated Rac1 controls Hh signaling in an Hh/Smo-independent manner.

To figure out the mechanisms underlying Rac1 affecting Hh signaling, we performed Gli-Luc reporter assays in C3H10T1/2 cells transfected with sgRNAs that targeting *Ptch1* or *SuFu*, the main negative regulators in Hh transduction 34,35. As expected, either *Ptch1* knockout or *SuFu* knockout significantly increased Gli-Luc activities (Figure 1J). Of note, although inhibition of Rac1 by NSC23766 significantly inhibited Gli-Luc activities in control cells (Con) and in the *Ptch1* knockout cells, it failed to inhibit the Gli-Luc activities induced by *SuFu*-deficiency (Figure 1J), suggesting Rac1 activates Hh signaling through inhibiting SuFu. Since activation of Hh causes release of Gli from SuFu-association, resulting in translocation of Gli from cytoplasm into the nucleus 36,37, we hypothesized that Rac1 may affect SuFu-Gli protein-protein complex formation. To test this idea, daRac1 was transfected into C3H10T1/2 cells and the cell lysates were subjected to immunoprecipitation with an anti-SuFu antibody. Our results revealed that SuFu bound to Gli1, while activation of Rac1 induced the dissociation of SuFu from its binding with Gli1 (Figure 1K-L). On the contray, inactivation of Rac1 by NSC23766 effectively strengthened this binding (Figure 1M-N). Similar results could be obtained by immunoprecipitation experiments of Gli2, showing Rac1 activation effectively disrupted the Gli2-SuFu complex formation (Figure 1O-P). Taken together, Rac1 activation promotes Hh signaling by controlling the dissociation of Gli and SuFu.

### Hh regulates Rac1 activity and Rac1 through PAK1 mediates Hh signaling

**Figure 2:**
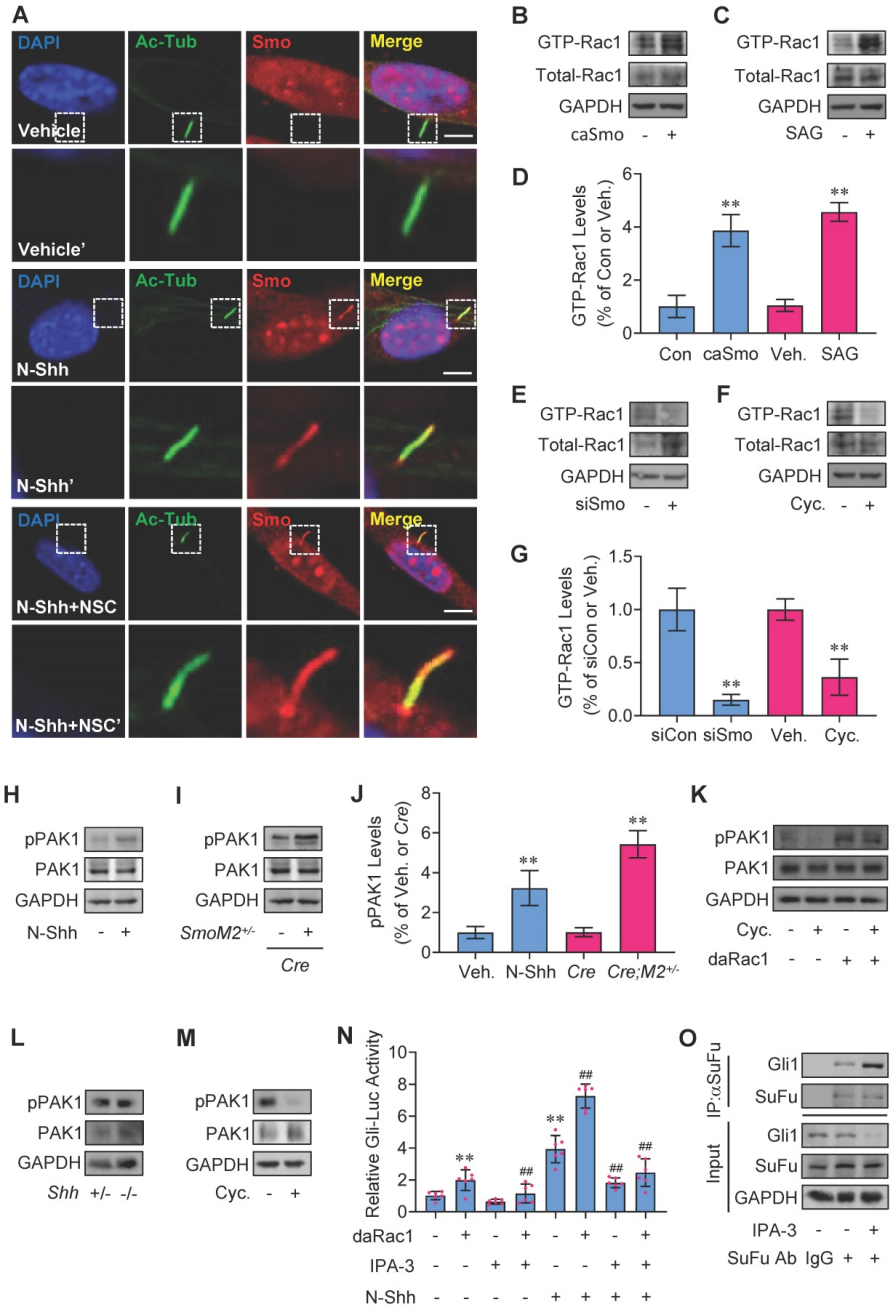
** Rac1 activation by Hh and regulation of Rac1-mediated Hh via PAK1. (A)** Immunofluorescence staining for Smo in MEFs cultured with or without N-Shh at 100 ng/ml for 48 h and NSC23766 (NSC) at 10 μg/ml for 24 h. Primary cilia were indicated by Ac-Tub staining. Nuclei were counterstained by DAPI. Bar, 20 μm. **(B,C)** Rac1 activation assays in C3H10T1/2 cells transfected with caSmo for 24 h (B) or cultured with SAG at 50 nM for 24 h (C). **(D)** Quantification via densitometry (n=3) and statistical analysis of GTP-Rac1 bands of (B) and (C). **(E,F)** Rac1 activation assays in C3H10T1/2 cells transfected with siSmo (E) for 72 h or cultured with Cyclopamine (Cyc., F) at 5 μM for 24 h. **(G)** Quantification via densitometry (n=3) and statistical analysis of GTP-Rac1 bands of (E) and (F). **(H)** Immunoblotting analyses of phospho-PAK1 (pPAK1) and PAK1 in C3H10T1/2 cells cultured with or without N-Shh at 100 ng/ml for 24 h. **(I)** Immunoblotting analyses of pPAK1 and PAK1 in cerebellum tissues of *GFAP-Cre;SmoM2^+/-^* and *SmoM2^+/-^* mice. **(J)** Quantification via densitometry (n=3) and statistical analysis of pPAK1 bands of (H) and (I). **(K)** Immunoblotting analyses of pPAK1 and PAK1 in C3H10T1/2 cells transfected with or without daRac1 for 24 h and cultured with or without Cyclopamine (Cyc.) at 5 μM for 24 h. **(L)** Immunoblotting analyses of pPAK1 and PAK1 in isolated MEFs from *Shh^+/-^* and *Shh^-/-^* embryos. **(M)** Immunoblotting analyses of pPAK1 and PAK1 in isolated MEFs cultured with or without Cyclopamine (Cyc.) at 5 μM for 24 h. **(N)** C3H10T1/2 cells were transiently transfected with a Gli luciferase reporter together with the daRac1 and cultured with or without N-Shh at 100 ng/ml or IPA-3 at 1 μM for 24 h. Total cell lysates were subjected to luciferase assay. N=6. **(O)** C3H10T1/2 cells were cultured with or without IPA-3 at 1 μM for 24 h. Total cell lysates (Input) and anti-SuFu immunoprecipitates (IP, SuFu Ab, +) from total cell lysates were analyzed by immunoblotting with anti-SuFu and anti-Gli1 antibodies. Protein abundance normalized to GAPDH, respectively. **, ^##^*p* < 0.01; error bar, SD.

In agreement with the previous reports 38,39, binding of Shh to receptor Ptch1 triggers the activation of Smo with the dramatic increase in the localization of Smo to primary cilia, while inactivation of Rac1 did not affect the presence of Smo in MEF primary cilia but attenuated N-Shh- or caSmo-induced Gli-Luc activities in C3H10T1/2 cells (Figure 1F, H and 2A), suggesting Hh-Smo may regulate Rac1 activity. To next study the potentially reciprocal effect of Hh on Rac1 activity, we utilized an established binding assay as described previously 16 to determine whether the GTP-bound (active) forms of Rac1 were affected in response to Hh activation. Our results revealed that either overexpression of caSmo or treatment with the Smo agonist, SAG, significantly activated Rac1 over the control in C3H10T1/2 cells, respectively (Figure 2B-D). In contrast, either knockdown of Smo by transfection with Smo-siRNA or inhibition of Smo with cyclopamine (Cyc.) significantly reduced the Rac1 activity (Figure 2E-G). In addition, administraton of N-Shh increased the phosphorylation levels of PAK1 in C3H10T1/2 cells, a downstream effector of Rac1 (Figure 2H, J). This result was further verified in the medulloblastoma tissues from *GFAP-Cre;SmoM2^+/-^* mice, where constitutively active Smo (SmoM2) is expressed in cerebellar granule neuron precursors (GNPs) by using a human glial fibrillary acidic protein promoter-driven Cre (GFAP-Cre) (Figure 2I, J). Consistently, overexpression of daRac1 not only significantly induced the phosphorylation levels of PAK1, but also almost completely attenuated the cyclopamine (Cyc.) suppressed-phosphorylation levels of PAK1 (Figure 2K). On the other hand, *Shh*-deficiant (*Shh^-/-^*) embryos at E15.5 developed the phenotype with an accumulation of pigmented tissue at the base and just under the proboscis (Figure S2A), which was consistant with that in a previous report 40, while unexpectedly, in those MEFs (*Shh^-/-^*) the phosphorylation levels of PAK1 did not exhibit a significant decrease, compared with the control group (*Shh^+/-^*) (Figure 2L). We suppose that the constant phosphorylation levels of PAK1 in *Shh^-/-^* and *Shh^+/-^* MEFs might be due to the characteristics of MEFs that MEFs do not produce Shh ligands but instead respond to Shh ligands. Nevertheless, in agreement with the results from C3H10T1/2 cells, inhibition of Hh signaling by cyclopamine (Cyc.) down-regulated the phosphorylation levels of PAK1 in MEFs (Figure 2M). Thus, these data suggest the role of Rac1-PAK1 axis in Hh signaling regulation. To further confirm the requirement of PAK1 in Rac1-mediated Hh transduction, we performed experiments in the presence of IPA-3, a specific antagonist against PAK1. IPA-3 not only significantly restored the daRac1-induced Gli-Luc activities in the presence or absence of N-Shh, but also effectively promoted SuFu-Gli1 protein-protein complex formation (Figure 2N-O). In summary, Hh activates Rac1 and Rac1 regulates Hh signaling *via* PAK1.

### Hh-Smo modulates Rac1 activity through Vav2

**Figure 3:**
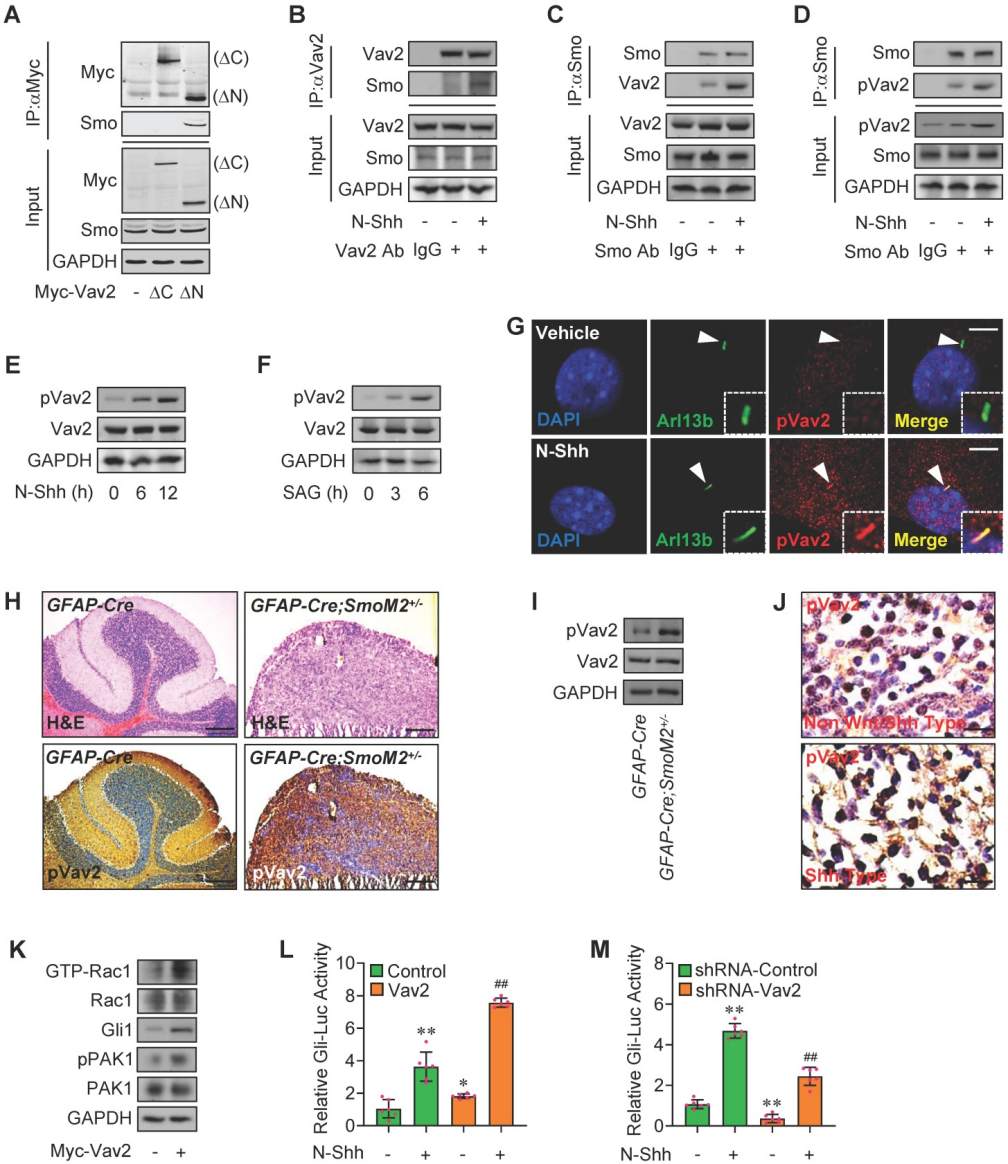
** Rac1 activation by Hh via Vav2. (A)** C3H10T1/2 cells were transiently transfected with the indicated plasmids. Total cell lysates (Input) and anti-Myc immunoprecipitates (IP) from total cell lysates were analyzed by immunoblotting with anti-Myc and anti-Smo antibodies. **(B)** C3H10T1/2 cells were cultured with or without N-Shh at 100 ng/ml for 24 h. Total cell lysates (Input) and anti-Vav2 immunoprecipitates (IP, Vav2 Ab, +) from total cell lysates were analyzed by immunoblotting with anti-Vav2 and anti-Smo antibodies. IgG was used as a negative control for IP. **(C)** C3H10T1/2 cells were cultured with or without N-Shh at 100 ng/ml for 24 h. Total cell lysates (Input) and anti-Smo immunoprecipitates (IP, Smo Ab, +) from total cell lysates were analyzed by immunoblotting with anti-Smo and anti-Vav2 antibodies. IgG was used as a negative control for IP. **(D)** C3H10T1/2 cells were cultured with or without N-Shh at 100 ng/ml for 24 h. Total cell lysates (Input) and anti-Smo immunoprecipitates (IP, Smo Ab, +) from total cell lysates were analyzed by immunoblotting with anti-Smo and anti-phospho-Vav2 (pVav2) antibodies. IgG was used as a negative control for IP. **(E)** Immunoblotting analyses of pVav2 and Vav2 in C3H10T1/2 cells cultured with or without N-Shh at 100 ng/ml for 0, 6 or 12 h. **(F)** Immunoblotting analyses of pVav2 and Vav2 in C3H10T1/2 cells cultured with or without SAG at 50 nM for 0, 3 or 6 h. **(G)** Immunofluorescence staining for pVav2 in MEFs with or without N-Shh at 100 ng/ml for 24 h. Primary cilia were indicated by Arl13b staining. Nuclei were counterstained by DAPI. Bar, 20 μm. **(H)** Hematoxylin-eosin (H&E) staining and immunohistochemistry staining for pVav2 in cerebella slides of *GFAP-Cre;SmoM2^+/-^* and *SmoM2^+/-^* mice. **(I)** Immunoblotting analyses of pVav2 and Vav2 in cerebella tissues of *GFAP-Cre;SmoM2^+/-^* and *SmoM2^+/-^* mice. **(J)** Immunohistochemistry staining for pVav2 in human clinical sample slides of non-Wnt/Shh-MB and Shh-MB. **(K)** Rac1 activation assays and immunoblotting analyses for Gli1, pPAK1 as well as PAK1 in C3H10T1/2 cells transfected with or without Myc-Vav2 for 24 h. **(L)** C3H10T1/2 cells were transiently transfected with a Gli luciferase reporter together with Vav2 and cultured with or without N-Shh at 100 ng/ml for 24 h. Total cell lysates were subjected to luciferase assay. N=6. **(M)** C3H10T1/2 cells were transiently transfected with a Gli luciferase reporter together with Vav2 shRNA and cultured with or without N-Shh at 100 ng/ml. Total cell lysates were subjected to luciferase assay. N=6. Protein abundance normalized to GAPDH, respectively. *p < 0.05; **, ^##^*p* < 0.01; error bar, SD.

Next, we wanted to know how Hh-Smo modulates Rac1 activity. To this end, we first tested the potential interaction of Smo and Rac1. Our results revealed that, although Smo bound to Rac1, Smo did not directly interact with Rac1, suggesting other molecule(s) involved in Smo-regulated Rac1 activity (Figure S3A-B). To further clarify the mechanisms underlying Rac1 activation by Hh-Smo, we examined several potential candidates that control Rac1 activity by Gli-Luc reporter assays, and finally we focused on Vav2, a guanine nucleotide exchange factor for Rac1 that contains a GEF domain in the N-terminus and a series of SH2-SH3-SH2 motifs in the C-terminus (Figure S3C, data not shown), and the interaction of Vav2 and Rac1 was then checked through the immunoprecipitation experiments with an anti-Vav2 antibody (Figure S3D). As expected, overexpression of full-length Vav2 (FL) obviously enhanced the SAG-induced Gli-Luc activities (Figure S3E), while interestingly, N-terminus of Vav2 (amino acid, aa 1-531, ΔC) showed little impact on Gli-Luc activities, whereas the C-terminus of Vav2 (aa 532-877, ΔN) revealed inhibitory effect to some extent on the SAG-induced Gli-Luc activities (Figure S3E). We speculated that the C-terminus of Vav2 may mainly serve as a scafold with the function of being bound by other molecules, and once upon bound to this region, the N-terminus with GEF domain would function as the switch that regulates the cycling between inactive GDP-bound and active GTP-bound forms of the Rho small GTPases such as Rac1. As expected, immunoprecipitation results revealed that the endogeneous Smo bound to the Myc-tagged Vav2 (Vav2 FL) as well as the Myc-tagged C-terminus of Vav2 (Myc-Vav2-ΔN) but not its N-terminus (Myc-Vav2-ΔC) in C3H10T1/2 cells (Figure 3A, Figure S3F). The endogeneous Smo-Vav2 protein-protein complex formation was further verified through immunofluorescence staining showing the well co-localization of endogeneous Smo and Vav2 in C3H10T1/2 cells (Figure S3G, upper), while unexpectedly but obviously, Smo-Vav2 complex formation was further enhanced in the presence of N-Shh, which was tested by immunoprecipitation experiments using either an anti-Vav2 antibody or an anti-Smo antibody (Figure 3B-C). Of note, the protein complexes precipitated with an anti-Smo antibody contained abundant endogenous Y172-phospho-Vav2, which was in consistence with the immunofluorescence staining data showing the co-localization of endogeneous Smo and Y172-phospho-Vav2 in C3H10T1/2 cells (Figure S3G, lower), and the complex formation between Smo and Y172-phospho-Vav2 was apparently strengthened by N-Shh stimulation (Figure 3D, IP), while the Vav2 phosphorylation levels at Y172 were acccordingly up-regulated in response to N-Shh (Figure 3D, Input), suggesting upon binding to Vav2, Smo induces Vav2 phosphorylation.

As expected, either N-Shh or SAG enhanced the phosphorylation levels of Vav2 at a time-dependent manner (Figure 3E-F), and the increased phosphorylation levels of Vav2 but not total Vav2 could be also observed at the primary cilia in MEFs as well as in C3H10T1/2 and JEG-3 cells (Figure 3G, Figure S3H-J). However, no significant change in phosphorylation levels of Vav2 was observed in HEK293T cells that lack primary cilia (Figure S3K), indicating the primary cilia are required for Hh-Smo-Vav2 signal. Moreover, the up-regulated phosphorylation levels of Vav2 were further verified in medulloblastoma tissues from *GFAP-Cre;SmoM2^ +/-^* mice as well as in human clinical Shh type MB samples (Figure 3H-J). While overexpression of Myc-tagged Vav2 (Myc-Vav2) apparently activated Rac1, potentiated Gli1 protein levels and PAK1 phosphorylation levels, and significantly induced Gli-Luc activities in the presence or absence of N-Shh (Figure 3K-L), knockdown of Vav2 attenuated the Gli-Luc activities induced by N-Shh (Figure 3M). Thus, the Hh-induced activated Smo binds to and phosphorylates Vav2 at Y172, resulting in the activation of Rac1 and thereby the phosphorylation of PAK1.

### Rac1 mediates Hh signaling by control of stabilization of IFT88

**Figure 4:**
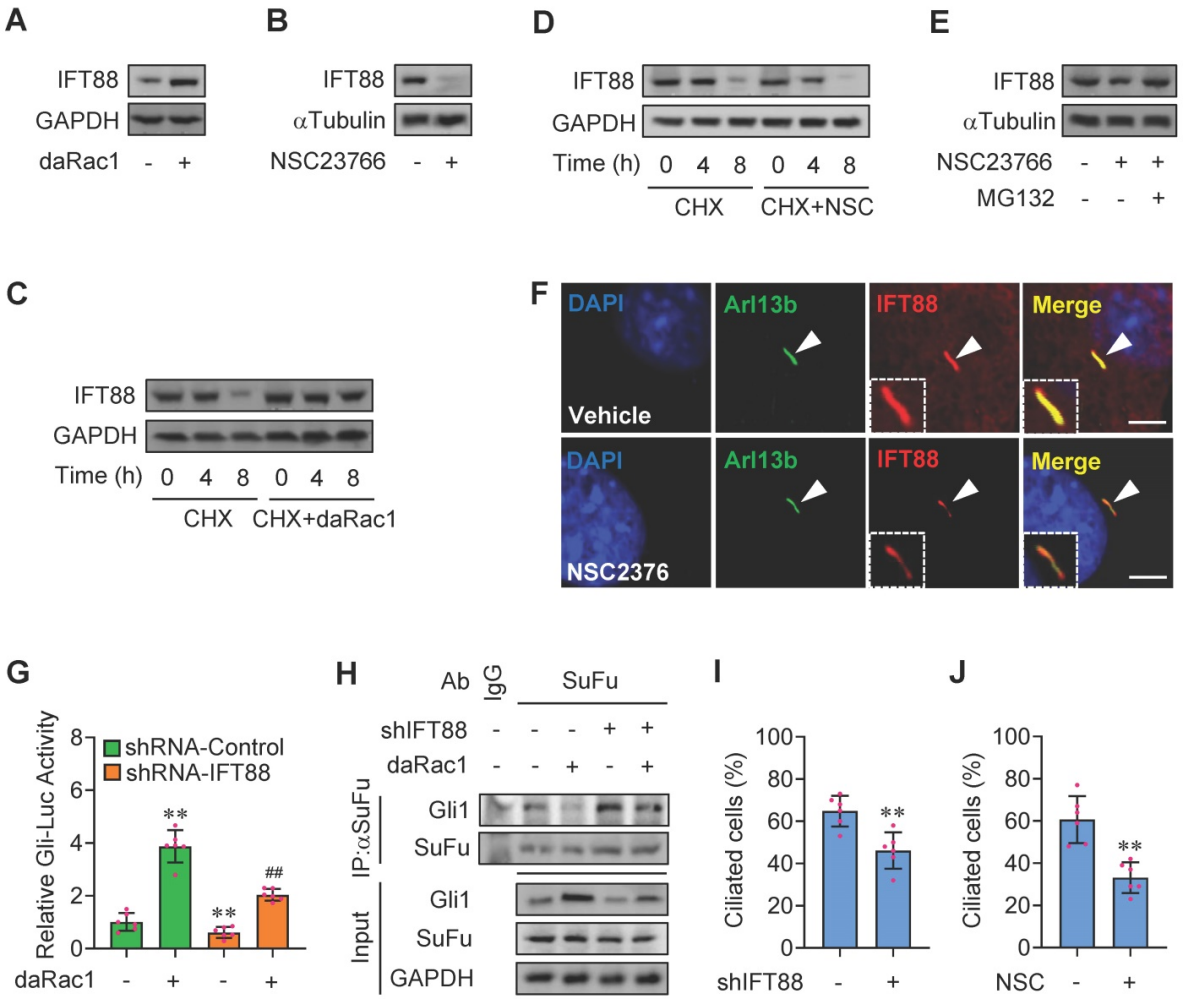
** Rac1 mediates Hh signaling by control of stabilization of IFT88. (A)** Immunoblotting analyses of IFT88 in C3H10T1/2 cells transfected with or without daRac1 and cultured for 24 h. **(B)** Immunoblotting analyses of IFT88 in C3H10T1/2 cells cultured with or without NSC23766 at 10 μg/ml for 24 h. **(C)** Immunoblotting analyses of IFT88 in C3H10T1/2 cells transfected with or without daRac1 and treated for different time periods with cycloheximide (CHX). **(D)** Immunoblotting analyses of IFT88 in C3H10T1/2 cells cultured with or without NSC23766 (NSC) at 10 μg/ml for 24 h and treated for different time periods with CHX. **(E)** Immunoblotting analyses of IFT88 in C3H10T1/2 cells cultured with or without NSC23766 at 10 μg/ml and MG132 at 10 μM for 24 h. **(F)** Immunofluorescence staining for IFT88 in MEFs with or without NSC23766 at 10 μg/ml for 24 h. Primary cilia were indicated by Arl13b staining. Nuclei were counterstained by DAPI. Bar, 20 μm. **(G)** C3H10T1/2cells were transiently transfected with a Gli luciferase reporter together with shIFT88 for 48 h and daRac1 for 24 h. Total cell lysates were subjected to luciferase assay. N=6. **(H)** C3H10T1/2 cells were transfected with shIFT88 and daRac1. Total cell lysates (Input) and anti-SuFu immunoprecipitates (IP) from total cell lysates were analyzed by immunoblotting with anti-Gli1 and anti-SuFu antibodies. IgG was used as a negative control for IP. **(I)** Statistical analysis for the percentage of ciliated C3H10T1/2 cells transfected with or without shIFT88. N=6. **(J)** Statistical analysis for the percentage of ciliated C3H10T1/2 cells cultured with or without NSC23766 (NSC) at 10 μg/ml for 24 h. N=6. Protein abundance normalized to GAPDH or αTubulin, respectively. **, ^##^*p* < 0.01; error bar, SD.

The primary cilium, a sensory and cell surface structure originating from a basal body derived from the mother centriole, is essential for Hh signaling transduction 41. The maintenance and elongation of cilia are mediated by a specialized bidirectional intraflagellar transport (IFT) system, including IFT-A and IFT-B, among which, IFT-B consists of IFT20, IFT52, IFT57, IFT88 subunits and IFT88 expresses most abundantly and is required for embryonic development 42,43. Western blot analyses in C3H10T1/2 cells revealed that overexpression of daRac1 significantly increased IFT88 protein levels, whereas Rac1 inactivation by NSC23766 obviously decreased IFT88 protein levels (Figure 4A-B). To further investigate the molecular mechanisms underlying IFT88 protein regulation by Rac1, we blocked *de novo* protein synthesis with cycloheximide (CHX) in C3H10T1/2 cells transfected with daRac1 or treated with NSC23766 (NSC) and analyzed the protein levels by western blot. As determined by the CHX chase assay in C3H10T1/2 cells, Rac1 activation extended the half-life of endogenous IFT88 protein, whereas Rac1 inactivation shortened the half-life of endogenous IFT88 protein, which could be effectively restored by the treatment of proteasome inhibitor MG132 (Figure 4C-E), and the decreased IFT88 protein levels were also observsed within primary cilia of the NSC23766-treated MEFs (Figure 4F), indicating Rac1 affects IFT88 protein stability.

To next test whether IFT88 is involved in Rac1-regulated Hh signaling, we constructed plasmids expressing *Ift88*-shRNAs which knocked down the expression of IFT88 by as much as 50-80% at either mRNA or protein levels (Figure S4A-B). Knockdown of IFT88 not only attenuated the daRac1-induced Gli-Luc activities and Gli1 protein levels, but also reversed the daRac1-disturbed Gli1-SuFu protein-protein complex formation (Figure 4G-H). Given IFT88 expression is associated with ciliogenesis 42, we additionally quatified the percentage of ciliated cells in the condition of Rac1 inactivation. Similar to the results of IFT88-knockdown, showing silence of IFT88 by shRNA significantly decreased the percentage of ciliated C3H10T1/2 cells, NSC23766 treatment gave rise to the significant down-regulation in the percentage of ciliated cells (Figure 4I-J). Thus, Rac1 activation promotes Hh signaling by stabilization of IFT88 protein.

### Phosphorylated KIF3A by Rac1 activation binds to and stabilizes IFT88 protein

**Figure 5:**
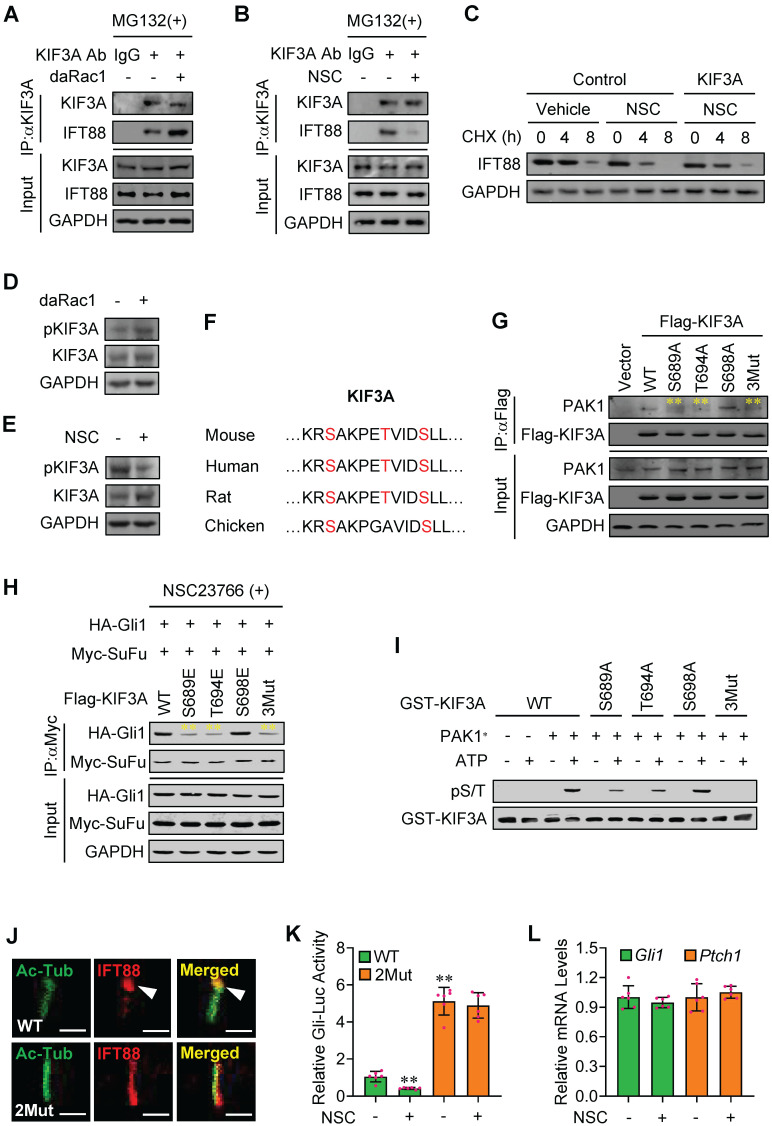
** Phosphorylated-KIF3A by Rac1 activation binds to and stabilizes IFT88 protein. (A)** C3H10T1/2 cells were transfected with or without daRac1 and cultured with MG132 at 10 μM. Total cell lysates (Input) and anti-KIF3A immunoprecipitates (IP, KIF3A Ab, +) from total cell lysates were analyzed by immunoblotting with anti-IFT88 and anti-KIF3A antibodies. IgG was used as a negative control for IP. **(B)** C3H10T1/2 cells were cultured with or without NSC23766 (NSC) at 10 μg/ml for 24 h and with MG132 at 10 μM. Total cell lysates (Input) and anti-KIF3A immunoprecipitates (IP, KIF3A Ab, +) from total cell lysates were analyzed by immunoblotting with anti-IFT88 and anti-KIF3A antibodies. IgG was used as a negative control for IP. **(C)** Immunoblotting analyses of IFT88 in C3H10T1/2 cells cultured with or without NSC23766 (NSC) at 10 μg/ml for 24 h and transfected with or without KIF3A for 24 h and treated for different time periods with CHX. **(D)** Immunoblotting analyses of phospho-KIF3A (pKIF3A, pSer/Thr) and KIF3A in C3H10T1/2 cells transfected with or without daRac1 for 24 h. **(E)** Immunoblotting analyses of pKIF3A and KIF3A in C3H10T1/2 cells cultured with or without NSC23766 (NSC) at 10 μg/ml for 24 h. **(F)** Conserved phosphorylation sites in KIF3A by PAK1 among species. **(G)** C3H10T1/2 cells were transfected with or without wild-type Flag-KIF3A (WT) or Flag-KIF3A mutant (S689A, T694A, S698A, 3Mut) and cultured for 48 h. Total cell lysates (Input) and anti-Flag immunoprecipitates (IP) from total cell lysates were analyzed by immunoblotting with anti-Flag and anti-PAK1 antibodies. **(H)** C3H10T1/2 cells were transfected with the indicated plasmids and cultured with NSC23766 at 10 μg/ml for 48 h. Total cell lysates (Input) and anti-Myc immunoprecipitates (IP) from total cell lysates were analyzed by immunoblotting with anti-Myc and anti-HA antibodies. **(I)** An *in vitro* kinase assay of GST-KIF3A and PAK1* in the presence or absence of ATP. Phospho-Ser/Thr (pS/T) was analysed by western blot. **(J)** Immunofluorescence staining for IFT88 in *Kif3a*-knockout C3H10T1/2 cells transfected with KIF3A (WT) or KIF3A mutant (2Mut, S689E + T694E) and cultured with NSC23766 at 10 μg/ml for 48 h. Primary cilia were indicated by Ac-Tub staining. Bar, 50 μm. **(K)** *Kif3a*-knockout C3H10T1/2 cells were transiently transfected with a Gli luciferase reporter together with wild type KIF3A (WT) or KIF3A mutant (2Mut, S689E + T694E) and cultured with or without NSC23766 (NSC) at 10 μg/ml for 48 h. Total cell lysates were subjected to luciferase assay. N=6. **(L)** *Kif3a*-knockout C3H10T1/2 cells were transiently transfected with KIF3A mutant (2Mut, S689E + T694E) and cultured with or without NSC23766 (NSC) at 10 μg/ml for 48 h. mRNA levels of *Gli1* and *Ptch1* were analyzed. N=6. RNA and protein abundance normalized to GAPDH, respectively. **p < 0.01; error bar, SD.

Movement of IFT-B along the cilia is powered by kinesin-II complex, among which KIF3A subunit plays the key role 44. Given that the protein levels of IFT88 were regulated by Rac1 activity (Figure 4C-E), we treated the cells with MG132 that inhibits proteasome-dependent IFT88 degradation and employed immunoprecipitation analysis. The protein complexes from C3H10T1/2 cells precipitated with a KIF3A antibody contained abundant endogenous IFT88, while this binding was strengthened by daRac1 (Figure 5A). In contrast, the KIF3A-IFT88 protein-protein complex formation was obviously impeded by Rac1 inactivation (Figure 5B).

To explore whether KIF3A participated in the Rac1-mediated Hh signaling, we constructed plasmids expressing *Kif3a*-shRNAs which knocked down the expression of KIF3A by as much as 40-80% at either mRNA or protein levels (Figure S5A-B). Overexpression of KIF3A not only partially restored the NSC23766 (NSC)-inhibited half-life of IFT88 protein (Figure 5C), but also effectively attenuated the NSC23766 (NSC)-decreased percentage of ciliated C3H10T1/2 cells (Figure S5C).

KIF3A activity is associated with its phosphorylation status 45,46. Western blot analysis revealed that daRac1 robustly up-regulated the phosphorylation levels of KIF3A, whereas Rac1 inactivation resulted in the apparently down-regulated phosphorylation levels of KIF3A (Figure 5D-E). To figure out the phospho-sites in KIF3A regulated by PAK1, we predicted the potential phosphorylational sites in KIF3A using BioGPS database (biogps.org) and obtained three candidates in its C-terminal, including S689, T694 and S698, which are highly conserved among species (Figure 5F). Mutation of S689A (Ser to Ala) or T694A (Thr to Ala) but not S698A significantly dampened the KIF3A binding to PAK1, whereas mutation of S689E (Ser to Glu) or T694E (Thr to Glu) but not S698E obviously impeded the NSC23766 induced-Myc-SuFu/HA-Gli1 protein-protein complex formation (Figure 5G-H). Moreover, triple muations of S689A, T694A and S698A (3Mut in Figure 5G) in KIF3A further suppressed its binding to PAK1, while triple muations of S689E, T694E and S698E (3Mut in Figure 5H) in KIF3A effectively reversed the NSC23766-induced Myc-SuFu/HA-Gli1 protein-protein complex formation (Figure 5G-H). Accordingly, an* in vitro* kinase assay consolidated the direct phosphorylation of KIF3A at S689 and T694 by PAK1 (Figure 5I). To check the functions of the clarified specific PAK1 phospho-sites in KIF3A in Hh signaling transduction, we additionally established *Kif3a*-knockout (KO) C3H10T1/2 cell line using CRISPR/Cas9 method. Our results exhibited that, though inactivation of Rac1 apparently dampened the turnover of IFT88 at ciliary tips and obviously inhibited Gli-Luc activities in wildtype KIF3A-transfected *Kif3a*-KO C3H10T1/2 cells (WT), it faild to do so in KIF3A mutant (S689E + T694E)-transfected *Kif3a*-KO cells (2Mut), which was in consistence with the qRT-PCT results of *Gli1* and *Ptch1* mRNA levels, showing the KIF3A mutant (S689E + T694E)-transfected *Kif3a*-KO cells (2Mut) exhibited little impact on the Gli-Luc activities in the presence or absence of NSC23766 (NSC) (Figure 5J-L). Thus, Rac1 activation by Hh stimulation gives rise to the phosphorylation of KIF3A at S689 and T694 through phosphorylated PAK1, resulting in the KIF3A binding to IFT88 and thereby the stabilization of IFT88 protein.

### *Rac1* is involved in Shh-MB progression and limb bud development

**Figure 6:**
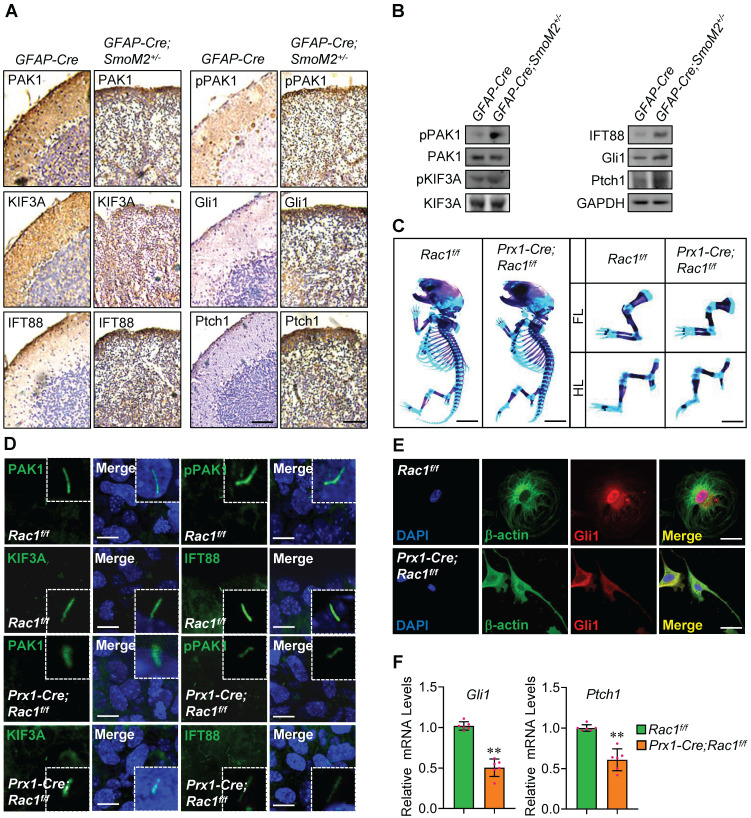
** Involvement of *Rac1* in Shh-MB and limb bud development. (A)** Immunohistochemistry staining for PAK1, pPAK1, IFT88, KIF3A, Gli1 and Ptch1 in cerebella tissue slides of *GFAP-Cre;SmoM2^+/-^* and *SmoM2^+/-^* mice. **(B)** Immunoblotting analyses of PAK1, pPAK1, IFT88, KIF3A, pKIF3A, Gli1 and Ptch1 in cerebella tissues of *GFAP-Cre;SmoM2^+/-^* and *SmoM2^+/-^* mice. **(C)** Skeletal preparations of the *Prx1-Cre;Rac1^f/f^* and *Rac1^f/f^* mice and their fore- (FL) and hindlimbs (HL) at postnatal day 0 (P0). **(D)** Immunofluorescence staining for PAK1, pPAK1, IFT88 and KIF3A in limb buds of *Prx1-Cre;Rac1^f/f^* and *Rac1^f/f^* mouse embryos at E10.5. Nuclei were counterstained by DAPI. Bar, 5 μm. **(E)** Immunofluorescence staining for Gli1 in primary mouse embryonic limb bud fibroblasts of *Prx1-Cre;Rac1^f/f^* and *Rac1^f/f^*. Cytoskeletons were stained by β-actin. Nuclei were counterstained by DAPI. Bar, 20 μm. **(F)** mRNA levels of *Gli1* (left) and *Ptch1* (right) in primary mouse embryonic limb bud fibroblasts of *Prx1-Cre;Rac1^f/f^* and *Rac1^f/f^*. N=6. RNA and protein abundance normalized to GAPDH, respectively. **p < 0.01; error bar, SD.

Activation of Smo in *GFAP-Cre;SmoM2^+/-^* mice developed medulloblastoma, showing severe disorganization in cerebella 47. To determine the physiological relevance of Rac1 in Hh signaling, we examined the key molecules in cerebella tissues from the *GFAP-Cre;SmoM2^+/-^* mice and the control *GFAP-Cre* mice. Immunohistochemistry data revealed that, although PAK1 and KIF3A protein levels did not present significant changes, the phosphorylation levels of PAK1 were apparently up-regulated, and IFT88 protein levels as well as the protein levels of targets, Ptch1 and Gli1, were obviously increased in cerebella tissues from *GFAP-Cre;SmoM2^+/-^* mice (Figure 6A), and the protein expression was further confirmed by western blot analysis (Figure 6B).

In agreement with a previous report, *Rac1* loss in mouse embryonic limb bud ectoderm (*Prx1-Cre;Rac1^f/f^*) had smaller skeletons than their *Rac1^f/f^* littermates with profound soft tissue syndactyly (Figure 6C) 48, indicating the crucial roles for *Rac1* in limb bud morphogenesis. Immunofluorescence staining in limb buds at E10.5 showed that, compared to those in *Rac1^f/f^*, the phosphorylation levels of PAK1 were significantly down-regulated and IFT88 protein levels were markedly decreased at the primary cilia in *Prx1-Cre;Rac1^f/f^* limb buds (Figure 6D), suggesting Rac1 regulation of Hh signaling at primary cilia as well *in vivo*. The knockout efficiency of *Rac1* was confirmed by immunofluorescence staining and western blot analysis (Figure S6A, B). Moreover, we found that the frequency of occurrence of nuclear accumulation of the transcriptional activators, Gli1 and Gli2, was decreased in the isolated mouse mesenchymal cells from *Prx1-Cre;Rac1^f/f^* limb buds, compared to that of *Rac1^f/f^* (Figure 6E, Figure S6C), and likewise, NSC23766 treatment apparently decreased the nuclear Gli1 protein levels in MEFs (Figure S6D). Consistently, the mRNA levels of the target genes, *Gli1* and *Ptch1*, were dramatically decreased in the isolated cells from *Prx1-Cre;Rac1^f/f^* limb buds (Figure 6F), demonstrating *Rac1* loss leads to the reduction in Hh signaling.

**Figure 7:**
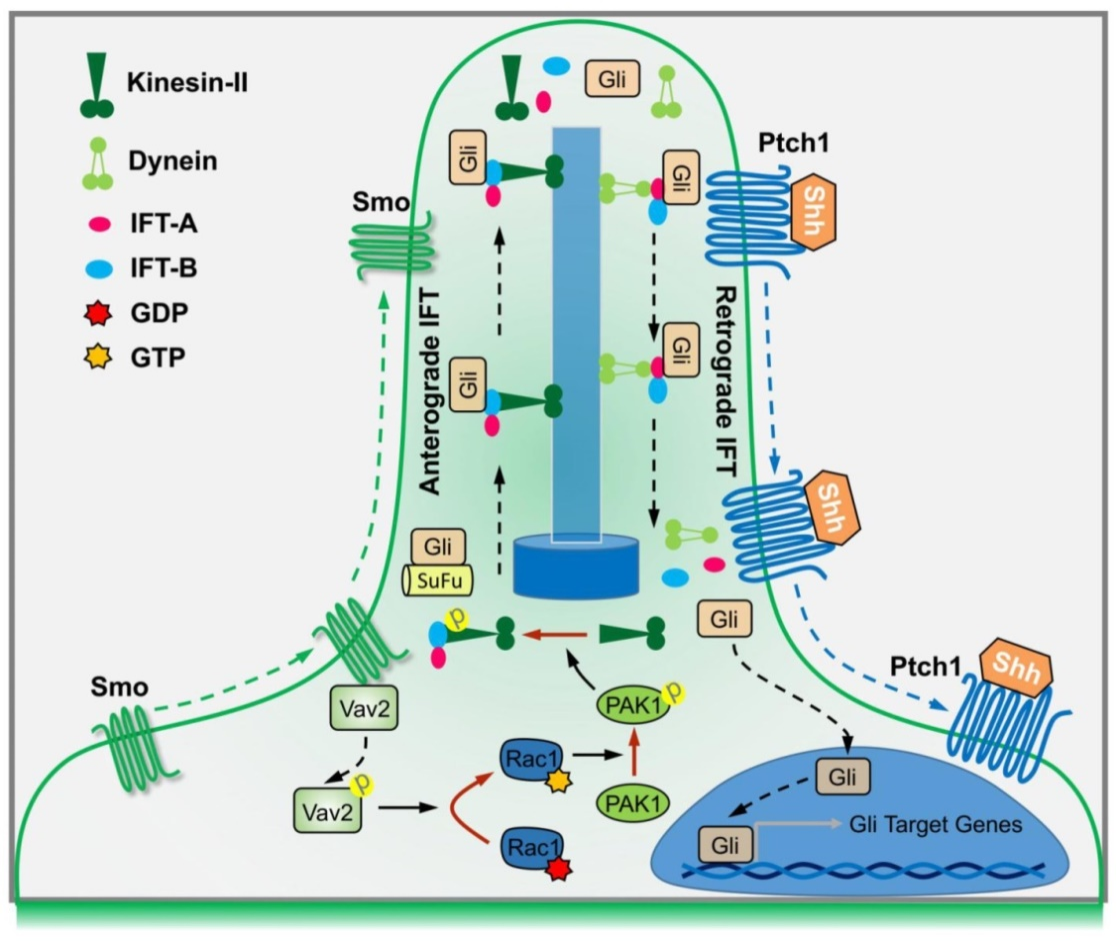
A summary working model

In summary, binding of Shh to Ptch1 triggers the ciliary enrichment of Smo. Simultaneously, Smo binds to and phosphorylates Vav2 at Y172, which further activates Rac1. The role of Rac1 is dependent on its regulation of phosphorylation levels of KIF3A through PAK1, which in turn binds to IFT88 and regulates IFT88 protein stability, and subsequently dampens the SuFu-Gli complex formation, resulting in the translocation of Gli into nucleus and transactivation of target genes (Figure 7).

## Discussion

By using biochemical, genetic and clinical approaches, we have uncovered a signaling cascade that operates in conjunction with Gli nuclear translocation to activate Hh signaling. Studies in C3H10T1/2 cells and MEFs support a model in which Hh signals through Smo to activate a signaling module composed of Vav2-Rac1-PAK1-KIF3A-IFT88-SuFu (Figure 7). As a result, Gli is released from its association with SuFu and thereby translocates into the nucleus. Our results are not only consistent with previous findings that Hh through primary cilia promotes Gli activation 11,49,50, but also have clarified the underlying mechanisms that Hh activation leads to Gli nuclear localization.

The Rho family of small GTPases is a key regulator of diverse cellular functions that impact cell growth, survival, motility, morphogenesis and differentiation 51,52. Members of the family, including RhoA, Rac1 and Cdc42, have been shown to participate in development through control of cellular signaling pathways, such as canonical Wnt signaling, non-canonical Wnt signaling, Notch signaling and Hippo signaling 16,53-55. Although the three members, RhoA, Rac1 and Cdc42, share some overlapping functions, RhoA and Cdc42 affected Hh signaling by other mechanisms (data not shown), which are different from our Rac1 study presented here.

Rac1 has been implicated in nuclear transport of other proteins. A previous study showed that nuclear accumulation of an armadillo protein SmgGDS is regulated by Rac1 activation 56, and, Kawashiwa *et al.* reported the regulation of nuclear translocation of STAT transcription factors through interactions with a GTPase-activating protein MgcRacGAP by Rac1 57. More recently, our group revealed that Rac1 activation controls nuclear localization of β-catenin during Wnt signaling 16. In addition to those previous studies that clarified the role of Rac1 activation in protein nuclear localization, the present study has identified a mechanism in which Rac1 activation by Hh stimulation gives rise to Gli nuclear localization through disturbance of protein complex of SuFu-Gli.

Previous findings have elucidated the functional cooperation between Hh signaling and Rac1 in cancer. Hyper-activation of Hh signaling has been shown to cause formation of cancers, such as BCC and MB, and mutations of Hh pathway components have been found both in familial and sporadic cases 58-61. On the other hand, high levels of Rac1 activation have been found in cancers, such as the colon cancer cells and breast cancer cells 62,63, suggesting Rac1 activation contributes to tumorigenesis. Consistently, in our study presented here, Hh promotes Rac1 activation in Shh type MB as well as in mouse embryonic development. Given the fact that loss of *Rac1* resulted in abnormal limb bud morphogenesis in mice, suggesting Rac1 is essential for development, we surmise that it is possible Rac1 functions with thresholds. In the case where Rac1 activity is beyond the lowest threshold, development is perturbed instead of tumoregenesis, whereas when Rac1 activity exceeds its highest threshold, cells are hyper-proliferated and thereby forming the tumor. Therefore, further experiments are needed to address these issues.

Primary cilia are often structurally adapted to serve diverse functions, such as signaling transduction functions by protein transportation within primary cilia. Observations from previous studies indicate the importance for a ciliary protein translocation system and IFT is thought to be the predominant pathway to move proteins into and along cilia, which proceeds bidirectional movements of supramolecular protein arrays inside cilia, including the movement from the basal body to the ciliary tip by anterograde IFT (IFT-B) and that of opposite direction by retrograde IFT (IFT-A) 12,64-66. IFT complexes move up and down the cilia using motor proteins, among which a heterotrimeric kinesin-2 powers IFT-B while dynein moves IFT-A. Cargo proteins of IFT, such as core components of the Hh signaling pathway, including Ptch1, Smo, SuFu as well as the Gli transcriptional factors, are localized to cilia. The final step of Hh signaling transduction is dependent on SuFu-Gli protein-protein complex dissociation. Once upon Hh activation, both SuFu and Gli proteins are picked up by IFT-B in the cell body, carried into cilia, released and dissociated in the cilia tips, and SuFu as well as Gli is moved back by IFT-A as a monomer, respectively. Our findings suggest that Rac1 activation contributes to IFT88 functions by regulation of its protein stabilization, resulting in the disassociation of SuFu-Gli complexes and the consequent release of Gli from SuFu, thereby the activation of Hh signaling. Nevertheless, whether other IFT-B subunits are affected by Rac1, and whether Rac1 regulates IFT-A as well are remained to be further determined.

Given the fact that Rac1-PAK1 activity is enhanced in MB tissue in *GFAP-Cre;SmoM2^+/-^* mice, further genetic studies are necessary to determine the role of *Rac1* in Hh signaling during other pathological settings besides the MB. In addition, it is of interest to determine whether *Rac1* hyper-activation could restore the phenotype caused by *Shh* deficiency (*Shh^-/-^*), such as that with only one fused forearm (zeugopod) bone and no digit arch (autopod) forms in developing limbs 40. The *Prx1-Cre;Rac1^f/f^* embryos give rise to limb bud defects to some extent, however, it is worth noting that the phenotype induction by *Rac1* deficiency is different from that in *Shh^-/-^* embryos. Given that the precise regulation of limb bud development is simultaneously controlled by other cellular signalings such as the Wnt/β-catenin pathway, which directs the embryonic limb apical ectodermal ridge (AER) formation thereby promoting limb outgrowth, it is possible that such differences between *Rac1* loss and *Shh* deficiency could be due to the diverse role of *Rac1* in limb bud development. A previous report has shown that *Rac1* is involved also in the canonical Wnt signaling transduction, whose loss in the mouse embryonic limb bud ectoderm (*Msx2-Cre;Rac1^c/+^*) disrupts canonical Wnt signaling and phenocopies deletion of *β-catenin* (*Msx2-Cre; β-catenin^n/c^*) in causing severe truncations of the limb 16,67. While Hh signaling acts upstream of the Wnt signaling pathway and negatively regulates Wnt activity 68-70, the Wnt/β-catenin pathway reciprocally dampens Shh signaling activity and, intriguingly 71-73, Wnt activation is able to rescue the suppressive signaling of Hh in regulating amphibian limb regeneration 74, indicating that the imbalance of Hh and Wnt regulation serves a crucial role during embryogenesis including the developmental progression of limb buds. Thus, it will be interesting to further elucidate the mechanistic details whereby *Rac1* integrates Hh signaling and Wnt/β-catenin signaling during limb bud development *in vivo*.

Overall, our current study has substantiated the critical roles of Rac1 in Hh signaling transduction, and the Hh-Vav2-Rac1-PAK1 pathway may provide additional therapeutic targets for cancer cells of Hh activation.

## Materials & Methods

### Cell Lines

Mouse C3H10T1/2 cells, human embryonic kidney 293T (HEK293T) cells and human trophoblast-like JEG-3 cells were obtained from ATCC (Manassas, VA). The C3H10T1/2 cells, HEK293T cells and MEFs were cultured in high glucose DMEM (Life Technologies, Inc., Grand Island, NY) supplemented with 10% fetal bovine serum (FBS, Life Technologies, Inc., Grand Island, NY), 100 U/ml penicillin and 100 mg/ml streptomycin. JEG-3 cells were cultured in DMEM/F12 = 1:1 medium (Life Technologies, Inc., Grand Island, NY) supplemented with 10% (v/v) FBS (Life Technologies, Inc., Grand Island, NY), 100 U/ml penicillin and 100 mg/ml streptomycin. All the cells were maintained at 37 °C with 5% CO_2_.

### Oligonucleotides, Viruses and Infections

The primers for quantitative Real-time PCR (RT-PCR) were listed in the Supplement Data (Table S1). Lentiviruses expressing *Vav2*-shRNA, *Ift88*-shRNA, *Kif3a*-shRNA or *Rac1*-shRNA were generated by co-transfecting the HEK293T packaging cells with lentiviral shRNA expression vector, Pll3.7, inserted with the hairpin shRNA templates of complementary oligonucleotides (Table S2) at the sites of *XbaI* and *NotI*. Lentiviruses-containing supernatants with the titers greater than 1×10^6^ cfu/ml was used for infection of cells in the presence of 8 μg/ml polybrene (Sigma) as described previously 75.

### CRISPR/Cas9 Construction and Transfection

Expression vectors of sgRNA for mouse *Rac1*, *Ptch1*, *SuFu* or *Kif3a* were designed as pX330-based plasmids. Targeting sequences were designed using the CRISPR DESIGN tool (http://crispr.mit.edu/). All specific target sequences were amplified, cloned and verified by DNA sequencing. After the transient transfection of pX330-sg*Rac1*/*Ptch1*/*SuFu*/*Kif3a* plasmids together with a puromycin-resistant plasmid into cells by using Lipofectamine reagent (Invitrogen), puromycin (2 μg/ml, Invitrogen) treatment for 7 d was employed for selection and then cells were expanded in the regular culture medium.

### Antibodies, Proteins and Chemicals

Antibodies for PAK1, phospho-PAK1 and phospho-Ser/Thr were from Cell Signaling Technology; IFT88, KIF3A, SuFu, c-Myc, Gli1, acetylated-αTubulin (Ac-Tub), Vav2, GAPDH and β-actin antibdies were from Santa Cruz Biotechnology; Ptch1 antibody, Smo antibody and phospho-Vav2 antibody were from Abcam; Gli2 antibody and Rac1 antibody were from Bioss (Beijing, China); Rac2 and Rac3 antibodies were purchased from Huabio (Hangzhou, China); Flag-tag and HA-tag antibodies were from Beyotime (Shanghai, China); GST-tag antibody was from Yeason (Shanghai, China), and antibody anti-Arl13b was from Proteintech. Alexa555- and Alexa-488 conjugated secondary antibodies were from Life Technology. Recombinant mouse Shh protein (N-Shh, C25II, N-Terminus) was from R&D Systems (#464-SH). Cyclopamine, SAG and NSC23766 were purchased from Selleckchem. Cycloheximide and MG132 were from Beyotime (Shanghai, China).

### Luciferase Reporter Assays

Luciferase activities were measured using the dual luciferase reporter assay (Promega) according to the manufacturer's protocol. Renilla reporter plasmid was used as a second reporter. The data were obtained by analyzing triplicated samples each prepared from three independent experiments. The pcDNA3 empty vector was used as a control plasmid.

### RNA Isolation and Quantitative Real-time PCR (qRT-PCR)

Total RNA was isolated from C3H10T1/2 cells, MEFs or mouse limb buds by using a TRIzol reagent (Takara Biotechnology, Dalian, China) according to the manufacturer's instructions. 5 μg total RNA in a volume of 20 μl was reversely transcribed by using SuperScript III reagent (Life Technologies) and the oligo-(deoxythymidine) primer with incubation at 42 °C for 1 h. After the termination of cDNA synthesis, mRNA levels of target genes were determined by qRT-PCR as described previously 76. The relative amounts of the mRNA levels of the target genes were normalized to the GAPDH levels, respectively, and the relative difference in mRNA levels was calculated by 2^-ΔΔCt^ method.

### Nucleo-cytoplasmic separation assay, Western blot and Immunoprecipitation

For nucleo-cytoplasmic separation assay, the nuclear and cytosolic fractions of MEFs were prepared as described previously 77. The membrane proteins were isolated using a commercial kit as per the manufacturer's instructions (Sangon Biotech, Shanghai, China). Western blot and immunoprecipitation were performed as described previously 78. Total protein extracts were prepared, and protein concentrations were determined by using a standard Bradford assay. 50 μg of total protein was subjected to SDS-PAGE followed by a transfer onto PVDF membranes (Millipore, Bedford, MA). Membranes were incubated with primary antibodies anti-Smo (ab236465, abcam), anti-pVav2 (ab86695, abcam), anti-Gli1 (sc-20687, Santa Cruz), anti-Gli2 (bs-11564R, Bioss), anti-Vav2 (sc-271442, Santa Cruz), anti-acetylated-αTubulin (sc-23950, Santa Cruz), anti-c-Myc(sc-40, Santa Cruz), anti-KIF3A (sc-376680, Santa Cruz), anti-SuFu (sc-10933, Santa Cruz), anti-IFT88 (sc-84318, Santa Cruz), anti-GAPDH (sc-32233, Santa Cruz), anti-β-actin (sc-47778, Santa Cruz), anti-PAK1(#2602, Cell Signaling Technology), anti-pPAK1 (#2601, Cell Signaling Technology), anti-Rac1 (bs-4186R, Bioss), anti-Rac2 (ER61092, Huabio), anti-Rac3 (ER61093, Huabio), anti-GST (#30901ES10, Yeasen), anti-Flag (AF0036, Beyotime), anti-HA (AF2305, Beyotime) and anti-phospho-Ser/Thr (#9631, Cell Signaling Technology) followed by incubation in secondary antibodies. For immunoprecipitation, cells were prepared in whole cell lysis buffer, and the lysates were immunoprecipitated with various antibodies followed by SDS-PAGE and immunoblotting. Immunosignals were developed by using the Enhanced Chemiluminescence System. National Institutes of Health Image software (ImageJ, http://rsb.info.nih.gov/ij/) was used to quantify the immunoreactive bands, and the normalized antigen signals were calculated from target protein-derived and GAPDH or αTubulin-derived signals.

### Rac1 activation pull-down assay

Rac1 pull-down assay was performed as per the manufacturer's instruction (Cell Signaling Technology, USA). Briefly, GST-PAK1-PBD fusion protein beads were used to enrich active GTP-bound RAC1 in C3H10T1/2 cells, which were bound to glutathione agarose beads. Following this, the beads were washed with the lysis buffer and subjected to SDS-PAGE and immunoblotting analysis using a specific Rac1 antibody. In the end, lysates were examined for total Rac1 by western blot as described above.

### Immunofluorescence

Immunofluorescence was performed on chamber slides (Nalge Nunc International, Naperville, IL). After rinsed in PBS, samples (cells or limb bud tissues) were fixed in ice-cold methanol and permeabilized with 0.1% Triton X-100 in PBS (PBST). After incubation with blocking buffer for 30 min at room temperature, samples were incubated with primary antibodies against Smo (ab236465, abcam), Gli1 (sc-20687, Santa Cruz), pVav2 (ab86695, abcam), Vav2 (sc-271442, Santa Cruz), acetylated-αTubulin (sc-23950, Santa Cruz), KIF3A (sc-376680, Santa Cruz), IFT88 (sc-84318, Santa Cruz), pPAK1 (#2601, Cell Signaling Technology), PAK1 (#2602, Cell Signaling Technology), Arl13b (17711-1-AP, Proteintech) or β-actin (sc-47778, Santa Cruz) overnight at 4 °C. After washing with PBST, samples were further incubated with Alexa 488-conjugated or 555-conjugated secondary antibody (Life Technology). The nuclei were counterstained with 6'-diamidino-2-phenylindole (DAPI), and immunostaining was analyzed by a laser scanning microscope (Zeiss).

### Preparation of Recombinant Proteins

BL21 (DE3) *E. coli* strain transformed by the pet28b/GST-KIF3A or pet28b/GST-Rac1 plasmid was cultured at 37 °C in LB medium with ampicillin. When OD600 reached 0.8, the recombinant protein was inducibly expressed at 18 °C with 100 mM IPTG for 13 h. The obtained* E. coli* was suspended in Ni-1 buffer [20 mM Tris-HCl (pH 8.0), 100 mM MgCl_2_, 10 mM imidazole, 0.3 mg/ml benzamidine] and then the soluble fraction, which was prepared through ultrasonication (Astrason Ultrasonic Processor XL2020), and was incubated with Ni-NTA agarose beads (QIAGEN) at 4 °C for 1 h. After washing with Ni-2 buffer (20 mM Tris-HCl (pH 8.0), 100 mM MgCl_2_, 20 mM imidazole), the recombinant KIF3A or Rac1 was eluted into Ni-3 buffer [20 mM Tris-HCl (pH 8.0), 100 mM MgCl_2_, 250 mM imidazole] (Ni-affinity column chromatography). The lysate obtained was incubated with Glutathione Sepharose 4B beads (GE Healthcare) for 1 h at 4 °C and then applied to GST-affinity column chromatography, using W1 buffer [25 mM Tris-HCl (pH 7.5), 150 mM NaCl, 10 mM β-mercaptoethanol, 1% Triton X-100] and W2 buffer [50 mM Tris-HCl (pH 8.0), 150 mM NaCl, 10 mM β-mercaptoethanol] for washing. The recombinant KIF3A or Rac1 was eluted into W2 buffer with 10 mM glutathione and then concentrated *via* ultrafiltration using Amicon Ultra (Millipore).

### *In Vitro* Kinase Assay

Recombinant GST-KIF3A (10 pmol) and PAK1 (1 pmol) were mixed and incubated at 30 °C for 10 min in 30 μl of kinase buffer (10 mM HEPES, 150 mM NaCl, 2.5 mM DTT, 0.01% Triton X-100, 10 mM MnCl_2_) with or without 200 mM ATP. The *in vitro* phosphorylation was terminated by adding the SDS sample buffer to the reaction mixture. The reaction products were loaded onto an SDS-PAGE gel and then analyzed through the immunoblotting using the indicated antibodies.

### Cycloheximide Chase Analysis

C3H10T1/2 cells were transfected with either a daRac1-overxpression plasmid or a control empty vector and cultured for 24 h, or treated with either NSC23766 or vehicle for 24 h. Then, the medium was removed and complete medium with 100 μg/ml cycloheximide (CHX) was added into each well. At the indicated time, cells were lysed, and cell lysates were prepared for western blot analysis of IFT88 described above.

### Immunohistochemistry

Freshly sampled human tissues, mouse tissues or cells were flushed with ice-cold PBS and fixed by incubation in 4% paraformaldehyde in PBS overnight at 4 °C. Fixed samples were dehydrated, embedded in paraffin, and sectioned. The sections were de-waxed and rehydrated. Endogenous peroxidase activity was blocked by incubation in 0.3% hydrogen peroxide in methanol for 30 min at room temperature. Antigen retrieval was performed by boiling for 15 min in citrate buffer pH 6.0 or Tris-EDTA pH 9.0. Tissues were incubated overnight at 4 °C with the following primary antibodies: anti-IFT88 (sc-84318, Santa Cruz), anti-Gli1 (ab49314, abcam), anti-Ptch1 (ab53715, abcam), anti-KIF3A (sc-376680, Santa Cruz), anti-pVav2 (ab86695, abcam), anti-PAK1 (#2602, Cell Signaling Technology) and anti-pPAK1 (#2601, Cell Signaling Technology). Staining was performed using the Zsbio kit (Beijing, China) according to the manufacturer's instructions.

### Mass Spectrometry

Mass spectrometry was performed as reported previously 79. Briefly, the samples were loaded on gel and ran at 110 V for 90 min. Then, the proteins were digested with trypsin from duplicated gel, and the resulting peptides were separated with liquid chromatography-tandem mass spectrometry (LC-MS/MS) using ESI-QUAD-TOF (electrospray ionization quadrupole time-of-flight) commercially by Shanghai Luming Biotechnology Company. The peptides were identified using the UniProt protein database for mouse.

### Mouse Strains

*Shh^+/-^*, *Prx1-Cre*, *Rac1^f/f^* mouse strains were obtained from Jackson Laboratory (Bar Harbor, ME). *GFAP-Cre* mouse strain was kindly provided by Dr. Duan Shumin (Zhejiang University, China) and *SmoM2^+/-^* mouse strain was a gift from Dr. Chen Jianquan (Soochow University, China). The whole-mount skeletal preparations for mice were based on methods as previously described 80. Ethical approval for the study was granted from the Ethics Committee of School of Medicine of Zhejiang University.

### Statistical Analysis

All the numerous data were expressed as mean ± SD, and were analyzed by one-way ANOVA and Tukey-Kramer multiple comparison test (SPSS 13.0J software; SPSS, Inc., Chicago, IL). Statistical significance was assessed at *p* < 0.05 and *p* < 0.01. Experiments were independently triplicated, and results were qualitatively identical. Representative experiments are shown.

## Supplementary Material

Supplementary figures and tables.

## Abbreviations

Ab: antibody
Ac-Tub: acetylated tubulin
AER: apical ectodermal ridge
BCC: basal cell carcinoma
CHX: cycloheximide
CK1: casein kinase 1
Dhh: Desert Hedgehog
ESI-QUAD-TOF: electrospray ionization quadrupole time-of-flight
FBS: fetal bovine serum
GFAP: glial fibrillary acidic protein
GNPs: granule neuron precursors
GSK3β: glycogen synthase kinase 3β
HEK293T: human embryonic kidney 293T
Hh: Hedgehog
H&E: Hematoxylin-eosin
IFT: intraflagellar transport complexes
Ihh: Indian Hedgehog
KAP3: KIF associated protein 3
LC-MS/MS: liquid chromatography-tandem mass spectrometry
MB: medulloblastoma
MEFs: mouse embryonic fibroblasts
PKA: protein kinase A
Ptch1: Patched1
RT-PCR: Real-time PCR
Shh: Sonic Hedgehog
Smo: Smoothened
SuFu: Suppressor of Fused
