# Supplementary material for: Hedgehog signaling is controlled by Rac1 activity
Source: Theranostics. 2022 Jan 1;12(3):1303–20. doi: 10.7150/thno.67702 (PMC8771550; doi:10.7150/thno.67702)
Supplement: Supplementary file 1 — Supplementary figures and tables. [file thnov12p1303s1.pdf]

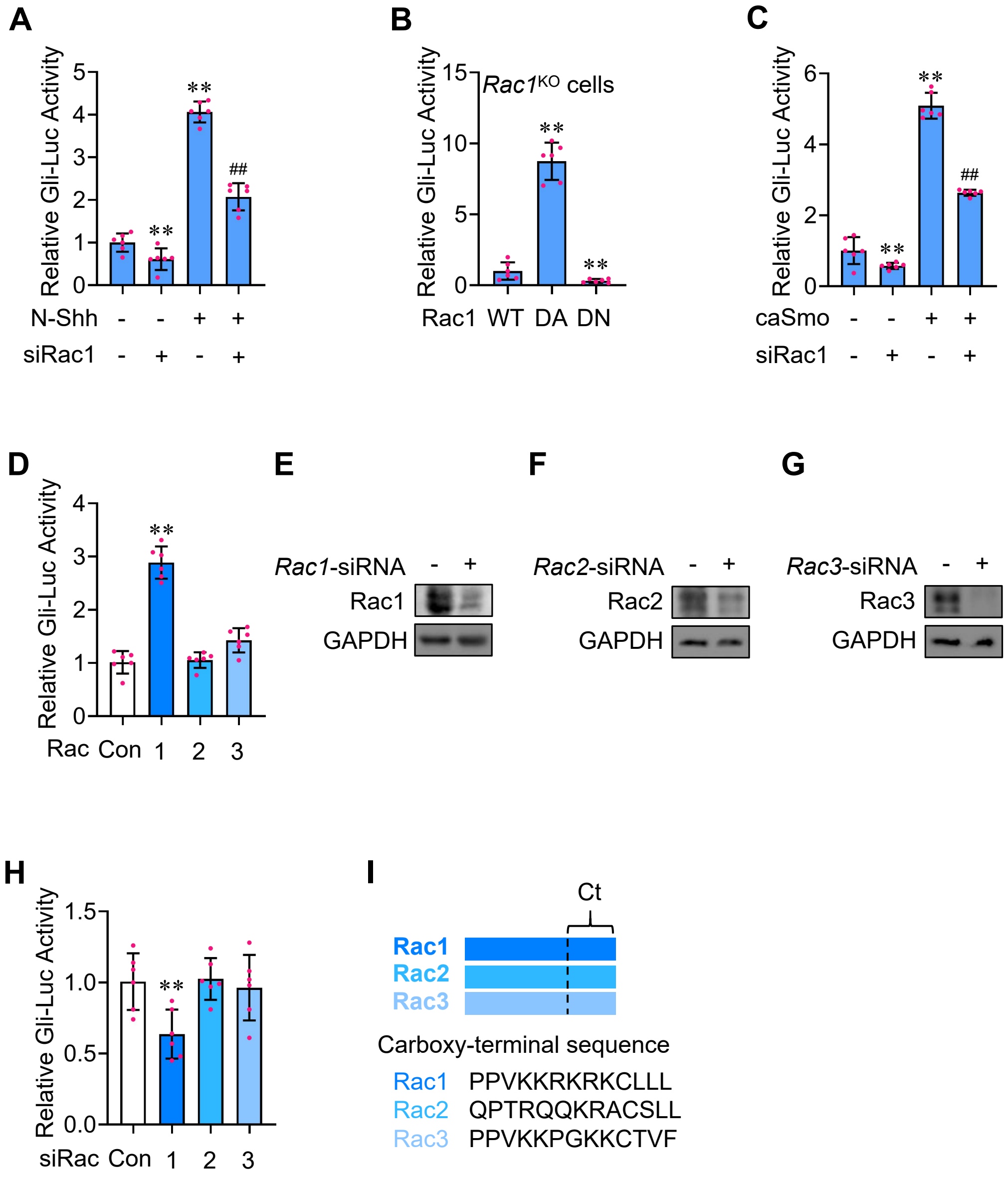
A
Relative Gli-Luc Activity
N-Shh - - + +
siRac1 - + - +
B
Rac1KO cells
Rac1 WT DA DN
C
caSmo - - + +
siRac1 - + - +
D
Rac Con 1 2 3
E
Rac1-siRNA - +
Rac1
GAPDH
F
Rac2-siRNA - +
Rac2
GAPDH
G
Rac3-siRNA - +
Rac3
GAPDH
H
siRac Con 1 2 3
I
Ct
Rac1
Rac2
Rac3
Carboxy-terminal sequence
Rac1 PPVKKRKRKCLLL
Rac2 QPTRQQKRACSLL
Rac3 PPVKKPGKKCTVF

Figure S1

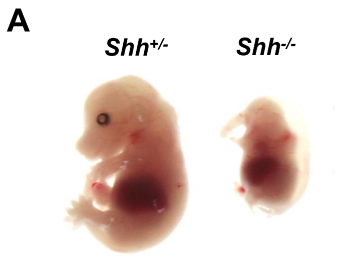

Figure S2

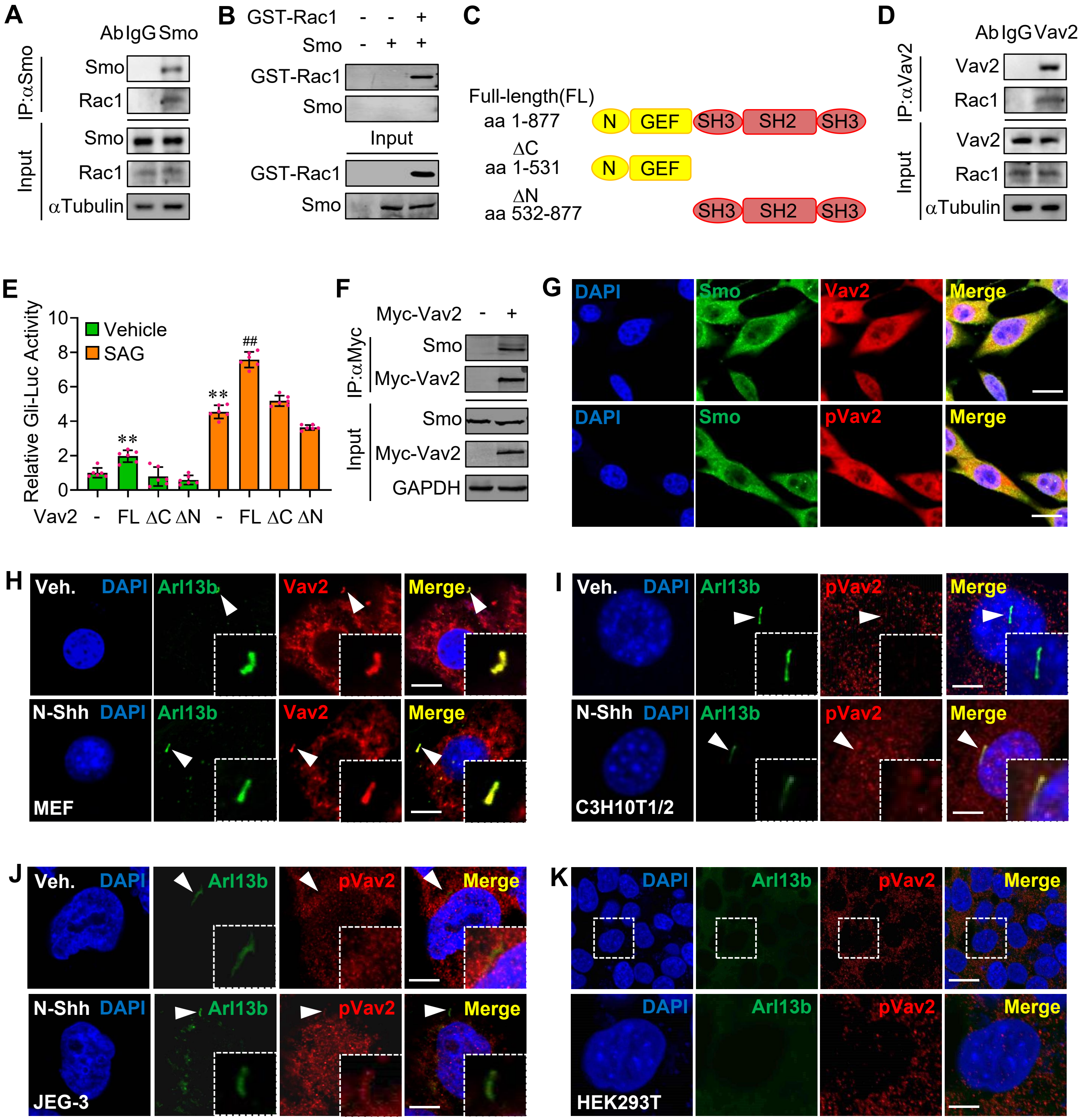

A
Ab IgG Smo
IP:αSmo
Smo
Rac1
Input
Smo
Rac1
αTubulin
B
GST-Rac1 - - +
Smo - + +
GST-Rac1
Smo
Input
GST-Rac1
Smo
C
Full-length(FL)
aa 1-877
N GEF SH3 SH2 SH3
ΔC
aa 1-531
N GEF
ΔN
aa 532-877
SH3 SH2 SH3
D
Ab IgG Vav2
IP:αVav2
Vav2
Rac1
Input
Vav2
Rac1
αTubulin
E
Relative Gli-Luc Activity
Vehicle
SAG
**
**
##
Vav2 - FL ΔC ΔN - FL ΔC ΔN
F
Myc-Vav2 - +
IP:αMyc
Smo
Myc-Vav2
Input
Smo
Myc-Vav2
GAPDH
G
DAPI Smo Vav2 Merge
DAPI Smo pVav2 Merge
H
Veh. DAPI Arl13b Vav2 Merge
N-Shh DAPI Arl13b Vav2 Merge
MEF
I
Veh. DAPI Arl13b pVav2 Merge
N-Shh DAPI Arl13b pVav2 Merge
C3H10T1/2
J
Veh. DAPI Arl13b pVav2 Merge
N-Shh DAPI Arl13b pVav2 Merge
JEG-3
K
DAPI Arl13b pVav2 Merge
DAPI Arl13b pVav2 Merge
HEK293T

Figure S3

A

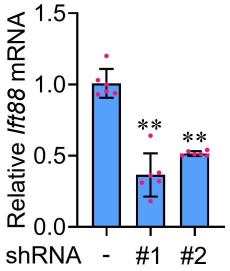

B

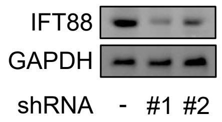

Figure S4

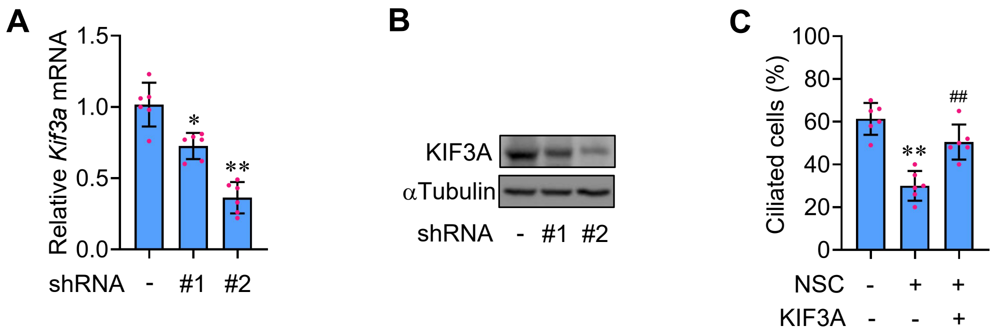
A
Relative Kif3a mRNA
1.5
1.0
0.5
0.0
*
**
shRNA
-
#1
#2
B
KIF3A
αTubulin
shRNA
-
#1
#2
C
Ciliated cells (%)
100
80
60
40
20
0
**
##
NSC
-
+
+
KIF3A
-
-
+

Figure S5

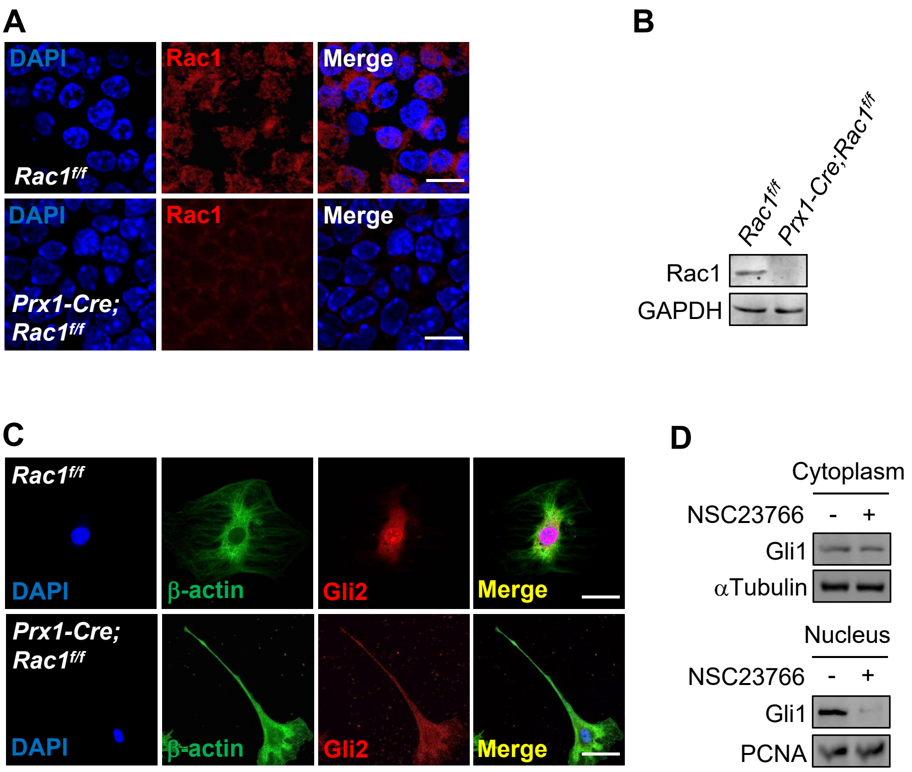
A
DAPI
Rac1
Merge
Rac1f/f
Prx1-Cre;
Rac1f/f
B
Rac1f/f
Prx1-Cre;Rac1f/f
Rac1
GAPDH
C
DAPI
β-actin
Gli2
Merge
D
Cytoplasm
NSC23766
- +
Gli1
αTubulin
Nucleus
PCNA

Figure S6

Table S1 The primers for quantitative RT-PCR assays

	Forward (5'-3')	Reverse (5'-3')
Gli1	CCAAGCCAACTTTATGTCAGGG	AGCCCGCTTCTTTGTTAATTTGA
Ptch1	CGGCAGCCGCGATAAG	TTAATGATGCCATCTGCATCCA
Vav2	GTTTGACCCTTTCGACCTCTTT	ACACAGTCGTAGATGTCCTCA
Ift88	TGGCCAACGACCTGGAGATTAACA	ATAGCTGCTGGCTTGGGCAAATTC
Kif3a	GAACAAGGTATCATCCCAAGGG	GCAGTGCCAACTTCTAGCAGA
Gapdh	TGGCCTTCCGTGTTCCTAC	GAGTTGCTGTTGAAGTCGCA

Table S2 The sequences for generating shRNA-expresing constructs

Sense (5'-3')	Antisense (5'-3')
***Vav2* nt 240**	
CCGGTCCGGCTGAAAGTCTGCCACGATAAAC TCGAGTTTATCGTGGCAGACTTTCAGTTTTTG	AATTCAAAAACTGAAAGTCTGCCACGATAAAC TCGAGTTTATCGTGGCAGACTTTCAGCCGGA
***Vav2* nt 1095**	
CCGGTCCGGTTGGCGATGTACATCAATGAAC TCGAGTTCATTGATGTACATCGCCAATTTTTG	AATTCAAAAATTGGCGATGTACATCAATGAAC TCGAGTTCATTGATGTACATCGCCAACCGGA
***Ift88* nt 813**	
CCGGTCCGGGCCAAATAAGTCATTTACCAAC TCGAGTTGGTAAATGACTTATTTGGCTTTTTG	AATTCAAAAAGCCAAATAAGTCATTTACCAAC TCGAGTTGGTAAATGACTTATTTGGCCCGGA
***Ift88* nt 2381**	
CCGGTCCGGGCCCTCAGATAGAAAGACCAA CTCGAGTTGGTCTTTCTATCTGAGGGCTTTTT G	AATTCAAAAAGCCCTCAGATAGAAAGACCAAC TCGAGTTGGTCTTTCTATCTGAGGGCCCGGA
***Kif3a* nt 698**	
CCGGTCCGGTGGAGTTTATGAATCAGCTAAC TCGAGTTAGCTGATTCATAAACTCCATTTTTG	AATTCAAAAATGGAGTTTATGAATCAGCTAAC TCGAGTTAGCTGATTCATAAACTCCACCGGA
***Kif3a* nt 1051**	
CCGGTCCGGAGGGATCAGAATAAATACAAAC TCGAGTTTGTATTTATTCTGATCCCTTTTTTG	AATTCAAAAAAGGGATCAGAATAAATACAAAC TCGAGTTTGTATTTATTCTGATCCCTCCGGA
